# Design, Synthesis, Antifungal Activities and 3D-QSAR of New *N*,*N*′-Diacylhydrazines Containing 2,4-Dichlorophenoxy Moiety

**DOI:** 10.3390/ijms141121741

**Published:** 2013-11-01

**Authors:** Na-Bo Sun, Yan-Xia Shi, Xing-Hai Liu, Yi Ma, Cheng-Xia Tan, Jian-Quan Weng, Jian-Zhong Jin, Bao-Ju Li

**Affiliations:** 1 College of Biology and Environmental Engineering, Zhejiang Shuren University, Hangzhou 310015, China; E-Mails: nabosun@gmail.com (N.-B.S.); jinjianzhongshuren@gmail.com (J.-Z.J.); 2 Institute of Vegetables and Flowers, Chinese Academy of Agricultural Sciences, Beijing 100081, China; E-Mail: shiyanxia813@163.com; 3 College of Chemical Engineering and Materials Science, Zhejiang University of Technology, Hangzhou 310014, China; E-Mails: tanchengxia@zjut.edu.cn (C.-X.T.); jqweng@zjut.edu.cn (J.-Q.W.); 4 State-Key Laboratory of Elemento-Organic Chemistry, National Pesticidal Engineering Centre, Nankai University, Tianjin 300071, China; E-Mail: mayink@126.com

**Keywords:** *N,N*′-diacylhydrazines, antifungal activity, synthesis, three-dimensional quantitative structure-activity relationships (3D-QSAR)

## Abstract

A series of new *N*,*N*′-diacylhydrazine derivatives were designed and synthesized. Their structures were verified by ^1^H-NMR, mass spectra (MS) and elemental analysis. The antifungal activities of these *N*,*N*′-diacylhydrazines were evaluated. The bioassay results showed that most of these *N*,*N*′-diacylhydrazines showed excellent antifungal activities against *Cladosporium cucumerinum*, *Corynespora cassiicola*, *Sclerotinia sclerotiorum*, *Erysiphe cichoracearum*, and *Colletotrichum orbiculare in vivo*. The half maximal effective concentration (EC_50_) of one of the compounds was also determined, and found to be comparable with a commercial drug. To further investigate the structure–activity relationship, comparative molecular field analysis (CoMFA) was performed on the basis of antifungal activity data. Both the steric and electronic field distributions of CoMFA are in good agreement in this study.

## 1. Introduction

**Figure 1:**
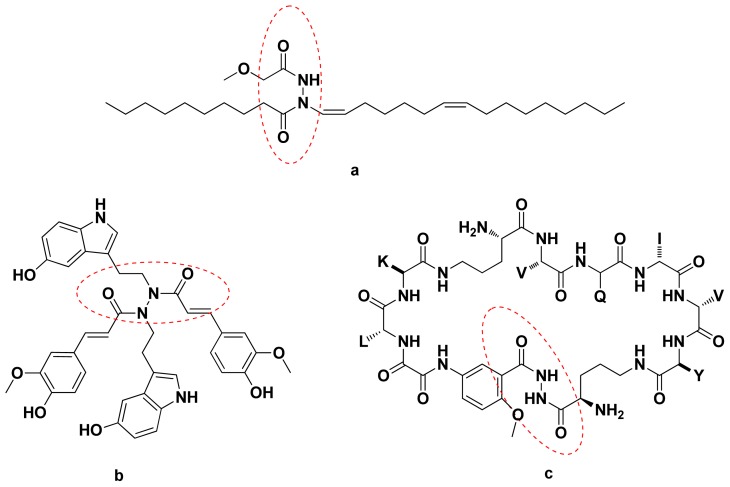
The natural products containing acylhydrazine structures.

In recent years, many diacylhydrazines have been isolated from natural products and displayed good biological activities. For example, Elaiomycin {[(2*S*,3*S*)-3-hydroxy-1-methoxybutan-2-yl]-imino-oct-1-enyloxidoazanium} (Figure 1a) is a natural compound isolated from submerged culture filtrates of *Streptomyces gelaticus*; it exhibits strong *in vitro* inhibition of virulent and avirulent forms of the bovine and human strains of *Mycobacterium tuberculosis* [1]. Montamine (Figure 1b), isolated from *C. montana*, is a new natural product that exhibits anti-oxidation activity [2]. Macrocyclic β-sheet peptides (Figure 1c) inhibit the aggregation of a tau-protein-derived hexapeptide [3]. Also diacylhydrazines are a class of insect growth regulators that have been found to act as nonsteroidal ecdysone agonists [4]. On the other hand, many synthetic diacylhydrazine compounds also exhibit a broad spectrum of biological activities, such as anti-HIV [5], fungicidal [6], ketol-acid reductoisomerase inhibitor (KARI) [6], plant growth regulatory activity [7], *etc.* 2,4-Dichlorophenoxyacetic acid (2,4-D) and their derivatives also exhibit many biological activities, such as antifungal activities [8], herbicidal activities [9], plant growth regulation activities [10] and so on.

There has been a tremendous increase in the frequency of fungal infections during the past four decades. Due to azoles exhibiting a broad antifungal spectrum, high potency and low toxicity, they are effective commercial antifungals for the treatment of these types of infections, and include: Fluconazole, Itraconazole, Miconazole, Econazole, Ketoconazole, Diniconazole, Triadimefon, Triadimenol, Flusilazole and so on. However, use of azoles have been limited because of the recent emergence of drug resistance. Hence, there is still a need to screen for safe and efficient antifungal agents with potent antifungal activities.

Quantitative structure-activity relationships (QSAR) play a vital role in modern drug design [11–13]. The modern QSAR method was found by Hansch and Fujita [14] and Free and Wilson [15] in 1964. The classical QSAR methods are 2D-QSAR and 3D-QSAR, because they allow rapid generation of QSARs from which biological activity of newly designed molecules can be predicted. Many QSAR methods had been developed, such as catastrophe QSAR [16], Alert-QSAR [17], Quantum-SAR [18], QSInAR [19], Hologram QSAR [20], Residual-QSAR [21] and so on. Currently, a new 2D-QSAR method, using Simplified Molecular Input Line Entry System (SMILES) molecules [22,23], will provide new insight in unfolding the necessary mechanism picture of a QSAR study in accordance with the Organization for Economic Co-Operation and Development (OECD) criteria[24].

In a previous paper, we reported some amide derivatives showed good antifungal activities [25–33]. In view of these facts, new diacylhydrazines were synthesized and evaluated. The results indicated that some of these derivatives exhibited excellent antifungal activity among the title compounds. The comparative molecular field analysis (CoMFA) was done for the structure-activity relationships (SAR) analysis.

## 2. Results and Discussion

### 2.1. Synthesis and Spectrum

**Scheme 1:**
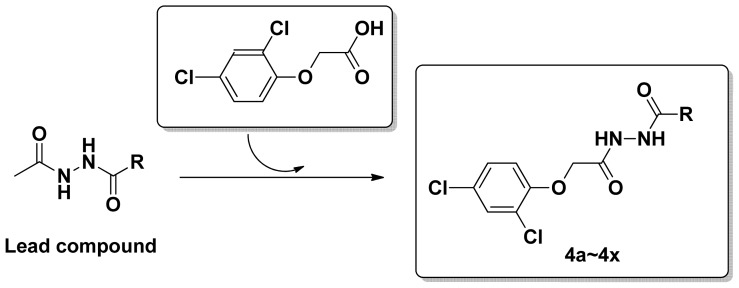
Design strategy of title compounds.

Referring to the reported references for function of diacylhydrazine derivatives, *N*,*N*′-diacylhydrazines are important insecticides in agriculture [34,35]. In our laboratory, we synthesized some diacylhydrazine derivatives successfully. They displayed good herbicidal activities [8], antifungal activities [6], radical scavenging activity [6], and plant growth regulatatory activity [8]. In order to find novel structural and non-resistance antifungals, the title compounds were designed by introducing 2-(2,4-dichlorophenoxy) acetic acid pharmacophore into the diacylhydrazine scaffold. Our original strategy is depicted in Scheme 1.

**Scheme 2:**
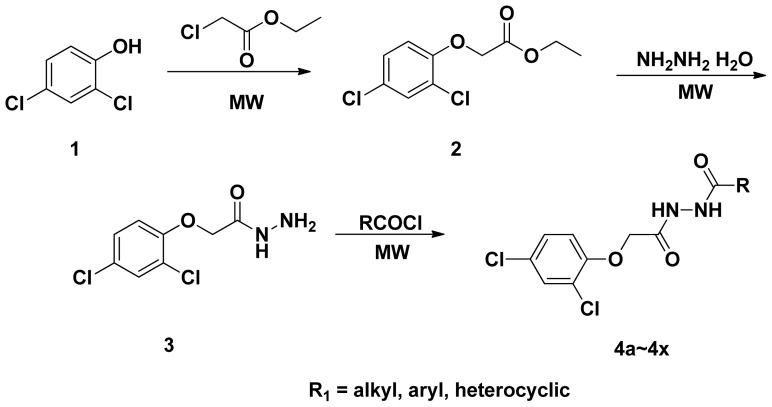
Synthetic route of title compounds.

The synthetic route of the title compounds were outlined in Scheme 2. 2-(2,4-Dichlorophenoxy) acetohydrazide was prepared from the starting material 2,4-dichlorophenol for 15 h. In order to optimize the reaction condition and reaction times, microwave irradiation was employed. The key intermediates **2** and **3** can be obtained with excellent yield (>95%) in a short reaction time. In the 1H-NMR spectra of title compounds, the CH_2_ proton signals of title compounds appeared at δ 4.57~4.82 ppm. The two NH proton was observed as two single peaks, and sometimes, it appeared as one broad peak. All the other alkyl or aryl groups showed the normal location. All the title compounds of mass spectra (MS) are molecular ion peak.

### 2.2. Antifungal Activities

The *in vivo* antifungal results of title compounds against *Cladosporium cucumerinum*, *Corynespora cassiicola*, *Sclerotinia sclerotiorum*, *Sphaerotheca fuligenea*, *Colletotrichum orbiculare* are listed in Table 1. Table 2 shows the EC_50_ value of the high fungicidal activity compound **4b**. Most of the compounds showed promising results in inhibiting the mycelial growth of all test fungi at a concentration of 500 μg/mL. Meanwhile, all of these compounds were found safe for the cucumber plants. As shown in Table 1, compound **4b**,**f**,**l**,**m** and **s** exhibited a significant inhibition effect against *Sphaerotheca fuligenea*, and the fungicidal activities (control efficacy of 77%–100%) were higher than that of control chlorothalonil. Compound **4b** particularly, exhibited good control effect (100%) against *Sphaerotheca fuligenea*. Compounds **4a**–**d**, **q** and **r** displayed a significant inhibition effect against *Colletotrichum orbiculare* and the antifungal activity (control effect 72%–98%) was higher than that of control. Compound **4f**, **g**, **j**–**m**, **t**, **u**, **w** and **x** showed moderate antifungal activity (control effect around 50%) against *Colletotrichum orbiculare*. Compounds **4p** and **q** possessed efficacy rates of 85% and 87% against *Corynespora cassiicola*, respectively. The two compounds were more effective than chlorothalonil. No fungicidal activities were found for these compounds against *Sclerotinia sclerotiorum*, except that compound **4f** (51.17%) and **4n** (55.13%) displayed a moderate control effect. For *Cladosporium cucumerinum*, it was found that **4d**, **h**, **k**, **q**, **t**, **u** and **x** exhibited moderate antifungal activity (control effect around 50%).

Table 1 shows that compound **4b** exhibited excellent antifungal activity against *Sphaerotheca fuligenea* and so the half maximal effective concentration (EC_50_) of **4b** was investigated. The EC_50_ results showed that compound **4b** had weaker activity against *Sphaerotheca fuligenea* than that of control flusilazole.

### 2.3. CoMFA Analysis

**Figure 2:**
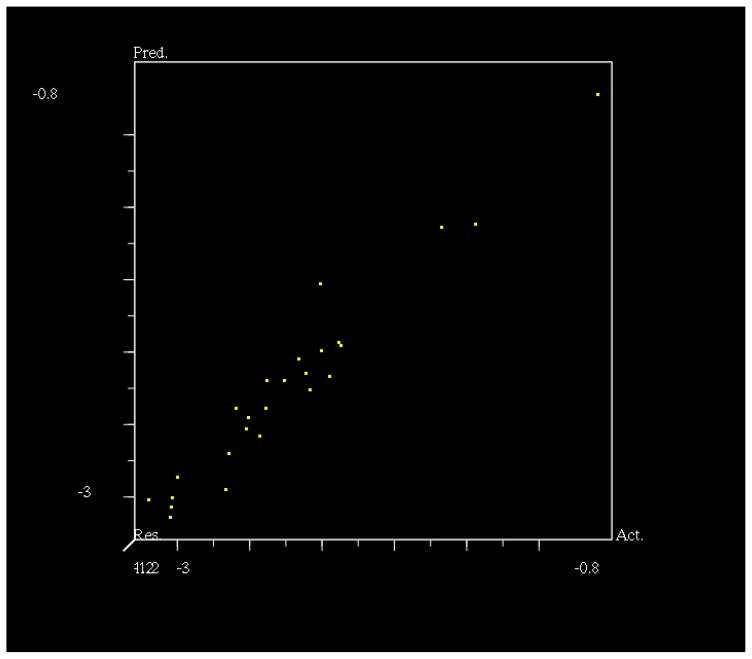
Comparative molecular field analysis (CoMFA) predicted as experimental −Log D (pD) values.

**Figure 3:**
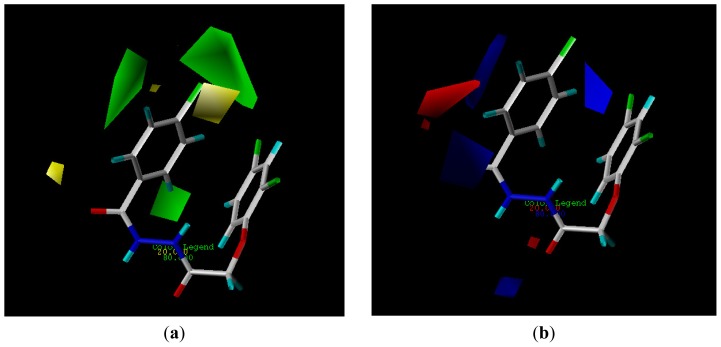
Steric and electrostatic contribution contour maps of CoMFA.

The CoMFA method is widely used in drug design, because it allows for rapid prediction of QSAR of newly designed molecules [36]. The CoMFA contour models are very similar, suggesting that for this training set, using four components is acceptable. Experimental and predicted activities by CoMFA for all compounds are listed in Table 3. As shown in Table 3, a predictive CoMFA model was established with the conventional correlation coefficient *r*^2^ = 0.858 and the cross-validated coefficient *q*^2^ = 0.61. It is shown in Figure 3 that the contributions of steric and electrostatic fields (“StDev*Coeff”) are 70.2% and 29.8% respectively. The plots of the predicted *vs* the actual activity values for all the compounds are shown in Figure 2.

With the CoMFA analysis, we obtained the isocontour diagrams of the steric and electrostatic field contributions (“StDev*Coeff”), which is displayed in Figure 3. In Figure 3a, the steric field contours are represented with different colors: the green color at 3- or 4-position means a bulky group here would be favorable for higher antifungal activity, while the yellow color means the opposite. As shown in Figure 3a, there is a green region located around the 4-position of the benzene ring, indicating that the bulky groups at this position will increase the herbicidal activity. This is in agreement with the actual experimental data: for example, compounds **4c**–**e**,**l**,**m** all have higher antifungal activity with a bulky group in this position. In the same Figure 3b, the electrostatic contours are displayed in distinguishable colors: blue indicates that an increase in the positive charge will lead to an increase in the activity, while the red contour defines the opposite. So, the target compounds bearing an electron-withdrawing group at the 2-position of the benzene ring and an electron-donating group at the other positions displayed higher activity. These results provided useful information for further optimization of the compounds.

## 3. Experimental Section

### 3.1. Instruments

Melting points were determined using an X-4 apparatus (Beijing Tech Instruments Co., Beijing, China) and uncorrected. ^1^H-NMR spectra were measured on a Bruker AC-P500 instrument (300MHz, Bruker, Fallanden, Switzerland) using Tetramethylsilane (TMS) as an internal standard and DMSO-*d*_6_ as solvent. Mass spectra were recorded on a Thermo Finnigan LCQ Advantage LC/mass detector instrument (ThermoFinnigan, Waltham, MA, USA). Elemental analyses were performed on a Vario EL elemental analyzer (Elementar, Hanau, Germany). All reagents are analytical grade or synthesized by ourselves.

### 3.2. General Procedure

2,4-Dichlorophenol (5 mmol), potassium iodide (KI) (1 mmol), *N*,*N*-dimethylformamide (DMF) (1 mL), ethyl 2-chloroacetate (5 mmol) and Tetrabutyl Ammonium Bromide (TBAB) (0.5 mmol) were placed in a dried round-bottomed flask, and the mixture was irradiated using microwaves (200 W) for 5 min. On completion of the reaction, the mixture was cooled to room temperature and then added to ethanol (10 mL) with constant stirring. After filtering off the inorganic salts, the reaction mixture was added to 85% hydrazine hydrate (5 mmol) and subjected to microwave irradiation (500 W) for an additional 2 min. Then, it was cooled to room temperature, allowed to settle for 1 h, and the precipitates were filtered off and recrystallised from ethanol to afford the pure product **3**. Then **3** (1 mmol) and substituted acyl chloride (1 mmol) were mixed in Tetrahydrofuran (THF). The mixture was put into the microwave oven (400 W) and irradiated for 10 min to produce the crude solid, which on recrystallization with ethanol gave the pure product as shown in Scheme 2.

#### *N*′-[2-(2,4-Dichlorophenoxy)acetyl]cyclopropanecarbohydrazide **4a**

White solid, yield 79%, m.p. 199–200 °C; ^1^H-NMR (DMSO-*d*_6_) δ: 0.58–0.78 (m, 4H, cyclopropane), 1.55–1.67 (m, 1H, cyclopropane), 4.70 (s, 2H, CH_2_O), 7.06 (d, *J* = 8.8 Hz, 1H, Ph), 7.35 (d, *J* = 8.9 Hz, 1H, Ph), 7.57 (s, 1H, Ph), 10.11 (s, 2H, NH); ESI-MS: 302.55 [M–H]^−^; Elemental analysis for C_12_H_12_Cl_2_N_2_O_3_: found C 47.45, H 4.08, N 9.31; calcd. C 47.54, H 3.99, N 9.24.

#### *N*′-[2-(2,4-Dichlorophenoxy)acetyl]benzohydrazide **4b**

White solid, yield 90%, m.p. 148–150 °C; ^1^H-NMR (DMSO-*d*_6_) δ: 4.80 (s, 2H, CH_2_O), 7.35–7.59 (m, 5H, Ph), 7.84–7.92 (m, 3H, Ph), 10.22 (s, 1H, NH), 10.47 (s, 1H, NH); ESI-MS: 338.54 [M–H]^−^; Elemental analysis for C_15_H_12_Cl_2_N_2_O_3_: found C 52.95, H 3.46, N 8.54; calcd. C 53.12, H 3.57, N 8.26.

#### *N*′-[2-(2,4-Dichlorophenoxy)acetyl]-4-nitrobenzohydrazide **4c**

White solid, yield 91%, m.p. 209–211 °C; ^1^H-NMR (DMSO-*d*_6_) δ: 4.82 (s, 2H, CH_2_O), 7.13 (d, *J* = 9.9 Hz, 1H, Ph), 7.36–7.40 (m, 1H, Ph), 7.59 (s, 1H, Ph), 8.00 (d, *J* = 8.3 Hz, 1H, Ph), 8.34 (d, *J* = 8.3 Hz, 1H, Ph), 10.39 (s, 1H, NH), 10.85 (s, 1H, NH); ESI-MS: 383.13 [M–H]^−^; Elemental analysis for C_15_H_11_Cl_2_N_3_O_5_: found C 47.02, H 3.11, N 11.12; calcd. C 46.90, H 2.89, N 10.94.

#### 4-Chloro-*N*′-[2-(2,4-dichlorophenoxy)acetyl]benzohydrazide **4d**

White solid, yield 88%, m.p. 199–201 °C; ^1^H-NMR (DMSO-*d*_6_) δ: 4.79 (s, 2H, CH_2_O), 7.14 (d, *J* = 8.9 Hz, 1H, Ph), 7.38 (d, *J* = 8.9 Hz, 1H, Ph), 7.55–7.61 (s, 3H, Ph), 7.86 (d, *J* = 8.6 Hz, 1H, Ph), 10.27 (s, 1H, NH), 10.57 (s, 1H, NH); ESI-MS: 371.93 [M–H]^−^; Elemental analysis for C_15_H_11_Cl_3_N_2_O_3_: found C 48.12, H 3.11, N 7.88; calcd. C 48.22, H 2.97, N 7.50.

#### *N*′-[2-(2,4-Dichlorophenoxy)acetyl]-3-methylbenzohydrazide **4e**

White solid, yield 82%, m.p. 160–162 °C; ^1^H-NMR (DMSO-*d*_6_) δ: 2.33 (s, 3H, CH_3_), 4.75 (s, 2H, CH_2_O), 7.14 (d, *J* = 8.9 Hz, 1H, Ph), 7.32–7.37 (m, 3H, ph), 7.32–7.37 (m, 3H, Ph), 7.54–7.62 (m, 2H, Ph), 7.66 (s, 1H, Ph), 10.34 (s, 2H, NH) ; ESI-MS: 352.65 [M–H]^−^; Elemental analysis for C_16_H_14_Cl_2_N_2_O_3_: found C 54.78, H 4.22, N 8.00; calcd. C 54.41, H 4.00, N 7.93.

#### 3-Chloro-*N*′-[2-(2,4-dichlorophenoxy)acetyl]benzohydrazide **4f**

White solid, yield 84%, m.p. 170–172 °C; ^1^H-NMR (DMSO-*d*_6_) δ: 4.76 (s, 2H, CH_2_O), 7.11 (d, *J* = 8.9 Hz, 1H, Ph), 7. 37 (d, *J* = 8.9 Hz, 1H, Ph), 7.40–7.59 (m, 3H, Ph), 7.80 (d, *J* = 8.9 Hz, 1H, Ph), 7.87 (s, 1H, Ph), 10.46 (s, 2H, NH); ESI-MS: 371.64 [M–H]^−^; Elemental analysis for C_15_H_11_Cl_3_N_2_O_3_: found C 48.45, H 2.78, N 7.33; calcd. C 48.22, H 2.97, N 7.50.

#### *N*′-[2-(2,4-Dichlorophenoxy)acetyl]-2-fluorobenzohydrazide **4g**

White solid, yield 81%, m.p. 148–150 °C; ^1^H-NMR (DMSO-*d*_6_) δ: 4.79 (s, 2H, CH_2_O), 7.12 (d, *J* = 8.9 Hz, 1H, Ph), 7. 27–7.38 (m, 3H, Ph), 7.53–7.61 (m, 3H, Ph), 10.33 (s, 2H, NH) ; ESI-MS: 356.44 [M–H]^−^; Elemental analysis for C_15_H_11_Cl_2_FN_2_O_3_: found C 50.56, H 3.33, N 8.02; calcd. C 50.44, H 3.10, N 7.84.

#### 2-Chloro-*N*′-[2-(2,4-dichlorophenoxy)acetyl]benzohydrazide **4h**

White solid, yield 90%, m.p. 186–188 °C; ^1^H-NMR (DMSO-*d*_6_) δ: 4.79(s, 2H, CH_2_O), 7.13 (d, *J* = 8.9 Hz, 1H, Ph), 7. 36 (d, *J* = 8.9 Hz, 1H, Ph), 7.41–7.50 (m, 4H, Ph), 7.59 (d, *J* = 2.6 Hz, 1H, Ph), 10.40 (s, 2H, NH); ESI-MS: 371.23 [M–H]^−^; Elemental analysis for C_15_H_11_Cl_3_N_2_O_3_: found C 48.44, H 3.12, N 7.78; calcd. C 48.22, H 2.97, N 7.50.

#### 2,4-Dichloro-*N*′-[2-(2,4-dichlorophenoxy)acetyl]benzohydrazide **4i**

White solid, yield 91%, m.p. 169–171 °C; ^1^H-NMR (DMSO-*d*_6_) δ: 4.77 (s, 2H, CH_2_O), 7.13 (d, *J* = 6.8 Hz, 1H, Ph), 7.34–7.39 (dd, *J* = 2.6 Hz, *J* = 2.6 Hz, 1H, Ph), 7.43–7.54 (m, 2H, Ph), 7.59 (d, *J* = 2.6 Hz, 1H, Ph), 7.71 (s, 1H, Ph), 10.48 (bs, 2H, NH); ESI-MS: 405.88 [M–H]^−^; Elemental analysis for C_15_H_10_Cl_4_N_2_O_3_: found C 44.33, H 2.44, N 7.09; calcd. C 44.15, H 2.47, N 6.86.

#### *N*′-[2-(2,4-Dichlorophenoxy)acetyl]-2-methoxybenzohydrazide **4j**

White solid, yield 99%, m.p. 175–177 °C; ^1^H-NMR (DMSO-*d*_6_) δ: 3.86 (s, 3H, OCH_3_), 4.76 (s, 2H, CH_2_O), 7.03 (t, *J* = 7.4 Hz, 1H, Ph), 7.14 (d, *J* = 7.8 Hz, 2H, Ph), 7.32–7.37 (m, 1H, Ph), 7.47–7.51 (m, 1H, Ph), 7.55–7.58 (m, 1H, Ph), 7.71 (d, *J* = 7.6 Hz, 1H, Ph), 10.26 (bs, 2H,NH); ESI-MS: 368.13 [M–H]^−^; Elemental analysis for C_16_H_14_Cl_2_N_2_O_4_: found C 51.95, H 4.08, N 7.89; calcd. C 52.05, H 3.82, N 7.59.

#### *N*′-[2-(2,4-Dichlorophenoxy)acetyl]-4-methoxybenzohydrazide **4k**

White solid, yield 79%, m.p. 174–175 °C; ^1^H-NMR (DMSO-*d*_6_) δ: 3.80 (s, 3H, OCH_3_), 4.78 (s, 2H, CH_2_O), 7.00 (d, *J* = 8.8 Hz, 2H, Ph), 7.14 (d, *J* = 8.9 Hz, 1H, Ph), 7.35 (d, *J* = 8.9 Hz, 1H, Ph), 7.58 (s, 1H, Ph), 7.83 (d, *J* = 8.8 Hz, 2H, Ph), 10.26 (s, 2H, NH) ; ESI-MS: 368.45 [M–H]^−^; Elemental analysis for C_16_H_14_Cl_2_N_2_O_4_: found C 52.11, H 4.02, N 7.87; calcd. C 52.05, H 3.82, N 7.59.

#### *N*′-[2-(2,4-Dichlorophenoxy)acetyl]-4-iodobenzohydrazide **4l**

White solid, yield 88%, m.p. 230–231 °C; ^1^H-NMR (DMSO-*d*_6_) δ: 4.79 (s, 2H, CH_2_O), 7.12 (d, *J* = 8.6 Hz, 1H, Ph), 7.35–7.39 (m, 1H, Ph), 7.59 (d, *J* = 8.9 Hz, 2H, Ph), 7.63 (s, 1H, Ph), 7.87 (d, *J* = 8.9 Hz, 2H, Ph), 10.26 (s, 1H, NH), 10.56 (s, 1H, NH); ESI-MS: 463.88 [M–H]^−^; Elemental analysis for C_15_H_11_Cl_2_IN_2_O_3_: found C 38.98, H 2.54, N 6.23; calcd. C 38.74, H 2.38, N 6.02.

#### *N*′-[2-(2,4-Dichlorophenoxy)acetyl]-5-methylisoxazole-4-carbohydrazide **4m**

White solid, yield 99%, m.p. 118–120 °C; ^1^H-NMR (DMSO-*d*_6_) δ: 2.62 (s, 3H, Het-CH_3_), 4.79 (s, 2H, CH_2_O), 7.10 (d, *J* = 8.9 Hz, 1H, Ph), 7.37 (d, *J* = 8.9 Hz, 1H, Ph), 7.58 (s, 1H, Ph), 8.90 (s, 1H, Het-CH), 10.27 (s, 1H, NH), 10.37 (s, 1H, NH); ESI-MS: 343.15 [M–H]^−^; Elemental analysis for C_13_H_11_Cl_2_N_3_O_4_: found C 45.66, H 3.56, N 12.31; calcd. C 45.37, H 3.22, N 12.21.

#### 1-Cyano-*N*′-[2-(2,4-dichlorophenoxy)acetyl]cyclopropanecarbohydrazide **4n**

White solid, yield 98%, m.p. 188–190 °C; ^1^H-NMR (DMSO-*d*_6_) δ: 1.51–1.64 (m, 4H, cyclopropane), 4.75 (s, 2H, CH_2_O), 7.00 (d, *J* = 6.0 Hz, 1H, Ph), 7.33 (d, *J* = 6.1 Hz, 1H, Ph), 7.57 (s, 1H, Ph), 10.31 (s, 2H, NH); ESI-MS: 327.66 [M–H]^−^; Elemental analysis for C_13_H_11_Cl_2_N_3_O_3_: found C 47.45, H 3.43, N 12.98; calcd. C 47.58, H 3.38, N 12.81.

#### *N*′-[2-(2,4-Dichlorophenoxy)acetyl] butyrohydrazide **4o**

White solid, yield 96%, m.p. 162–164 °C; ^1^H-NMR (DMSO-*d*_6_) δ: 0.84 (t, *J* = 7.3 Hz, 3H, CH_3_), 1.54 (q, *J* = 7.3 Hz, 2H, CH_2_), 2.00 (t, *J* = 7.2 Hz, 2H, CH_2_), 4.57 (s, 2H, CH_2_O), 7.05 (d, *J* = 8.9 Hz, 1H, Ph), 7.34 (d, *J* = 8.9 Hz, 1H, Ph), 7.56 (s, 1H, Ph), 9.23 (s, 1H, NH), 9.95 (s, 1H, NH); ESI-MS: 304.12 [M–H]^−^; Elemental analysis for C_12_H_14_Cl_2_N_2_O_3_: found C 47.44, H 4.78, N 9.23; calcd. C 47.23, H 4.62, N 9.18.

#### *N*′-[2-(2,4-Dichlorophenoxy)acetyl]isobutyrohydrazide **4p**

White solid, yield 92%, m.p. 174–176 °C; ^1^H-NMR (DMSO-*d*_6_) δ: 0.82 (d, *J* = 6.5 Hz, 6H, CH_3_), 1.99–2.11 (m, 1H, CH), 4.70 (s, 2H, CH_2_O), 7.01 (d, *J* = 9.0 Hz, 1H, Ph), 7.33 (d, *J* = 6.6 Hz, 1H, Ph), 7.56 (s, 1H, Ph), 9.97 (s, 2H, NH); ESI-MS: 304.95 [M–H]^−^; Elemental analysis for C_12_H_14_Cl_2_N_2_O_3_: found C 47.11, H 4.44, N 10.36; calcd. C, 47.23; H, 4.62; N, 9.18.

#### *N*′-[2-(2,4-Dichlorophenoxy)acetyl]butyrohydrazide **4q**

White solid, yield 96%, m.p. 162–164 °C; ^1^H-NMR (DMSO-*d*_6_) δ: 0.84 (t, *J* = 7.3 Hz, 3H, CH_3_), 1.54 (q, *J* = 7.3 Hz, 2H, CH_2_), 2.00 (t, *J* = 7.2 Hz, 2H, CH_2_), 4.57 (s, 2H, CH_2_O), 7.05 (d, *J* = 8.9 Hz, 1H, Ph), 7.34 (d, *J* = 8.9 Hz, 1H, Ph), 7.56 (s, 1H, Ph), 9.23 (s, 1H, NH), 9.95 (s, 1H, NH); ESI-MS: 304.12 [M–H]^−^; Elemental analysis for C_12_H_14_Cl_2_N_2_O_3_: found C 47.44, H 4.78, N 9.23; calcd. C 47.23, H 4.62, N 9.18.

#### *N*′-[2-(2,4-Dichlorophenoxy)acetyl]pentanehydrazide **4r**

White solid, yield 93%, m.p. 196–198 °C; ^1^H-NMR (DMSO-*d*_6_) δ: 0.85 (m, 3H, CH_3_), 1.48 (m, 4H, CH_2_), 2.08 (t, 2H, CH_2_), 4.81 (m, 2H, CH_2_O), 7.01 (d, 1H, Ph), 7.12 (d, 1H, Ph), 7.58 (d, 1H, Ph), 9.38 (s, 2H, NH); ESI-MS: 318.15 [M–H]^−^; Elemental analysis for C_13_H_16_Cl_2_N_2_O_3_: found C 49.21, H 5.22, N 9.01; calcd. C 48.92, H 5.05, N 8.78.

#### 2-(2,4-Dichlorophenoxy)-*N*′-[2-(2,4-dichlorophenoxy)acetyl]acetohydrazide **4s**

White solid, yield 92%, m.p. 214–216 °C; ^1^H-NMR (DMSO-*d*_6_) δ: 4.73 (s, 4H, CH_2_O), 7.01 (d, *J* = 9.0 Hz, 2H, Ph), 7.35 (d, *J* = 8.8 Hz, 2H, Ph), 7.58 (s, 2H, Ph), 10.26 (s, 2H, NH); ESI-MS: 437.95 [M–H]^−^; Elemental analysis for C_16_H_12_Cl_4_N_2_O_4_: found C 44.11, H 3.08, N 6.66; calcd. C 43.87, H 2.76, N 6.39.

#### 2-(2,4-Dichlorophenoxy)-*N*′-[2-(2,4-dichlorophenoxy)acetyl]propanehydrazide 4t

White solid, yield 96%, m.p. 245–246 °C; ^1^H-NMR (DMSO-*d*_6_) δ: 1.46 (d, *J* = 6.5 Hz, 6H, CH_3_), 4.80 (q, *J* = 6.5 Hz, 2H, Me-CH-OAr), 7.05 (d, *J* = 8.8 Hz, 2H, Ph), 7.29–7.32 (d, *J* = 8.9 Hz, 2H, Ph), 7.55 (s, 2H, Ph), 10.30 (bs, 2H, NH); ESI-MS: 465 [M–H]^−^; Elemental analysis for C_18_H_16_Cl_4_N_2_O_4_: found C 46.12, H 3.23, N 6.23; calcd. C 46.38, H 3.46, N 6.01.

#### *N*′-[2-(2,4-Dichlorophenoxy)acetyl]furan-3-carbohydrazide **4u**

White solid, yield 92%, m.p. 128–130 °C; ^1^H-NMR (DMSO-*d*_6_) δ: 4.72 (s, 2H, CH_2_O), 6.61 (s, 1H, Furan), 7.11–7.14 (m, 2H, Ph), 7.35 (m, *J* = 8.8Hz, 1H, Furan), 7.57 (s, 1H, Furan), 7.82 (s, 1H, Ph), 10.31 (s, 2H, NH); ESI-MS: 328.00 [M–H]^−^; Elemental analysis for C_13_H_10_Cl_2_N_2_O_4_: found C 44.54, H 3.29, N 8.24; calcd. C 44.74, H 3.06, N 8.51.

#### (2*E*,4*Z*)-*N*′-[2-(2,4-Dichlorophenoxy)acetyl]hexa-2,4-dienehydrazide **4v**

White solid, yield 98%, m.p. 132–134 °C; ^1^H-NMR (DMSO-*d*_6_) δ: 1.79 (d, *J* = 6.2 Hz, 3H, CH_3_), 4.73 (s, 2H, CH_2_O), 5.91 (d, *J* = 15.1 Hz, 1H, CH), 6.11–6.28 (m, 2H, CH), 7.03–7.14 (m, 2H, Ph), 7.35 (d, *J* = 8.9 Hz, 1H, Ph), 7.57 (s, 1H, Ph), 10.24 (s, 2H, NH) ; ESI-MS: 328.15 [M–H]^−^; Elemental analysis for C_14_H_14_Cl_2_N_2_O_3_: found C 50.95, H 4.44, N 8.88; calcd. C 51.08, H 4.29, N 8.51.

#### *N*′-[2-(2,4-Dichlorophenoxy)acetyl]nicotinohydrazide **4w**

White solid, yield 88%, m.p. 197–199 °C; ^1^H-NMR (DMSO-*d*_6_) δ: 4.81 (s, 2H, CH_2_O), 7.14 (d, *J* = 8.9 Hz, 1H, Ph), 7.51–7.54 (m, 1H, Py), 7.58(s, 1H, Ph), 8.19(d, *J* = 8.0 Hz, 1H, Py), 8.74 (d, *J* = 3.2 Hz, 1H, Py), 9.00(s, 1H, Py), 10.32(s, 1H, NH), 10.69(s, 1H, NH); ESI-MS: 339.56 [M–H]^−^; Elemental analysis for C_14_H_11_Cl_2_N_3_O_3_: found C 49.65, H 3.43, N 12.31; calcd. C 49.43, H 3.26, N 12.35.

#### *N*′-[2-(2,4-Dichlorophenoxy)acetyl]isonicotinohydrazide **4x**

White solid, yield 92%, m.p. 103–105 °C; ^1^H-NMR (DMSO-*d*_6_) δ: 4.80 (s, 2H, CH_2_O), 7.15 (d, *J* = 8.9 Hz, 1H, Ph), 7.38 (d, *J* = 8.9 Hz, 1H, Ph), 7.52 (s, 1H, Ph), 7.74 (d, *J* = 5.9 Hz, 2H, Py), 8.75 (d, *J* = 5.9 Hz, 2H, Py), 10.35 (s, 1H, NH), 10.78 (s, 1H, NH); ESI-MS: 339.15 [M–H]^−^; Elemental analysis for C_14_H_11_Cl_2_N_3_O_3_: found C 49.19, H 3.32, N 12.53; calcd. C 49.43, H 3.26, N 12.35.

### 3.3. 3D-QSAR Analysis

**Figure 4:**
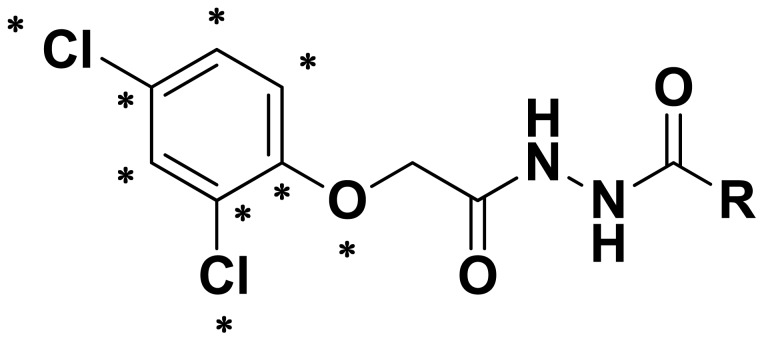
The asterisk skeleton of title compounds.

**Figure 5:**
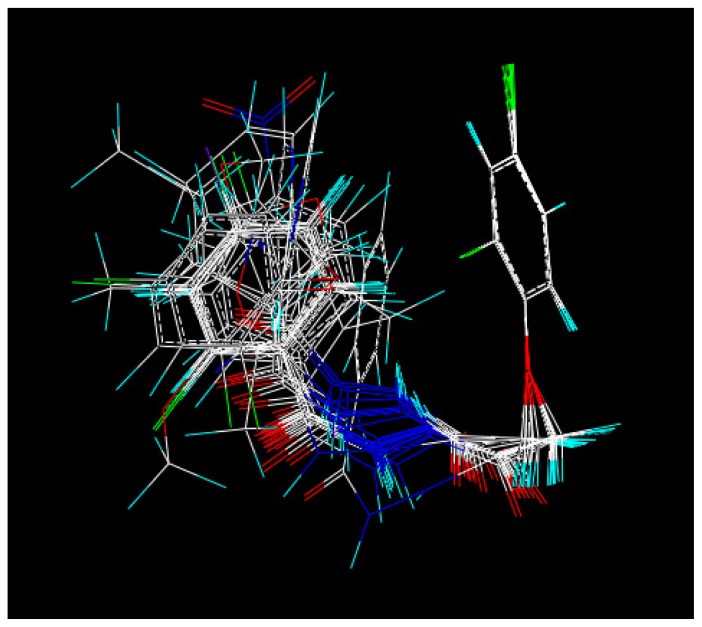
Superposition modes of compounds.

Molecular modeling was performed using SYBYL 6.91 software [36] (Tripos, Inc., St. Louis, MO, USA), and the CoMFA method according to our previous work [37]. The antifungal activities of 24 compounds against *Colletotrichum orbiculare* data (% I) at 500 μg/mL used to derive the CoMFA analyses model are listed in Table 3. The activity was expressed in terms of activity factor (D) by the formula:

(1) $$D=\text{log}\{I/[(100-I)\times M_{\text{w}}]\}$$

where *I* is the percent inhibition and *M*_w_ is the molecular weight of the tested compounds. The compound **4d** was used as a template to build the other molecular structures. Because these compounds share a common skeleton, 9 atoms marked with an asterisk were used for rms-fitting onto the corresponding atoms of the template structure (Figures 4 and 5).

Each structure was fully geometry-optimized using a conjugate gradient procedure based on the Tripos force field and Gasteiger and Hückel charges. Because these compounds share a common skeleton, 10 atoms marked with an asterisk were used for rms-fitting onto the corresponding atoms of the template structure. CoMFA steric and electrostatic interaction fields were calculated at each lattice intersection on a regularly spaced grid of 2.0 Å. The grid pattern was generated automatically by the SYBYL/CoMFA routine, and an sp^3^ carbon atom with a van der Waals radius of 1.52 Å and a +1.0 charge was used as the probe to calculate the steric (Lennard-Jones 6-12 potential) field energies and electrostatic (Coulombic potential) fields with a distance-dependent dielectric at each lattice point. Values of the steric and electrostatic fields were truncated at 30.0 kcal/mol. The CoMFA steric and electrostatic fields generated were scaled by the CoMFA-STD method in SYBYL. The electrostatic fields were ignored at the lattice points with maximal steric interactions. A partial least-squares (PLS) approach was used to derive the 3D-QSAR, in which the CoMFA descriptors were used as independent variables, and D values were used as dependent variables. The cross-validation with the leave-one-out (LOO) option and the SAMPLS program, rather than column filtering, was carried out to obtain the optimal number of components to be used in the final analysis. After the optimal number of components was determined, a non-cross-validated analysis was performed without column filtering. The modeling capability (goodness of fit) was judged by the correlation coefficient squared, *r*^2^, and the prediction capability (goodness of prediction) was indicated by the cross-validated *r*^2^ (*q*^2^).

### 3.4. Antifungal Activities Assay

Anti-fungal activity of compounds **4a**~**4x** against *Cladosporium cucumerinum*, *Corynespora cassiicola*, *Sclerotinia sclerotiorum*, *Sphaerotheca fuligenea*, *Colletotrichum orbiculare* were evaluated according to reference [38], and a potted plant test method was adopted. Germination was conducted by soaking cucumber seeds in water for 2 h at 50 °C and then keeping the seeds moist for 24 h at 28 °C in an incubator. When the radicles were 0.5 cm, the seeds were grown in plastic pots containing a 1:1 (*v*/*v*) mixture of vermiculite and peat. Cucumber plants used for inoculations were at the stage of two seed leaves. Tested compounds and commercial fungicides were sprayed with a hand spray on the surface of the seed leaves on a fine morning, at the standard concentration of 500 μg/mL. After 2 h, inoculations of *Cladosporium cucumerinum*, *Corynespora cassiicola*, *Sphaerotheca fuligenea*, *Colletotrichum orbiculare* were carried out by spraying a conidial suspension, and inoculation of *Sclerotinia sclerotiorum* was carried out by spraying a mycelial suspension. The experiment was repeated 4 times. After inoculation, the plants were maintained at 18–30 °C (mean temperature of 24 °C and above 80% relative humidity). Fungicidal activity was evaluated when the nontreated cucumber plant (blank) fully developed symptoms. The area of inoculated treated leaves covered by disease symptoms was assessed and compared to that of nontreated ones to determine the average disease index. The relative control efficacy of compounds compared to the blank assay was calculated via the following equation:

(2) relative control efficacy (%)=(CK-PT)/CK×100%

where *CK* is the average disease index during the blank assay and *PT* is the average disease index after treatment during testing.

## 4. Conclusions

In summary, a series of diacylhydrazine derivatives were synthesized containing a 2,4-dichlorophenoxy moiety in good yields. The preliminary bioassays showed that some of the compounds had good fungicidal activity. The structure–activity relationship, and CoMFA was performed. The present findings provided a powerful complement to the SARs of fungicides, and warrant future investigation of the mechanism of action of these analogues.

## Figures and Tables

**Table 1:** Antifungal activity of title compounds (percent relative control efficacy) at 500 μg/mL.

No.	*Corynespora cassiicola*	*Cladosporium cucumerinum*	*Sphaerotheca fuligenea*	*Sclerotinia sclerotiorum*	*Colletotrichum orbiculare*
**4a**	10.00	10.00	59.09	13.82	89.79
**4b**	49.00	20.00	100.00	15.55	72.52
**4c**	37.00	37.00	34.54	nd	72.77
**4d**	39.00	59.00	18.18	49.49	98.75
**4e**	42.00	4.00	42.70	42.23	68.70
**4f**	38.00	nd	77.60	51.17	46.48
**4g**	38.00	19.00	35.71	31.85	66.94
**4h**	38.00	71.00	67.27	16.14	19.87
**4i**	nd	nd	nd	−4.24	25.85
**4j**	42.00	7.00	12.34	13.97	43.18
**4k**	41.00	49.00	30.30	31.06	63.46
**4l**	35.00	13.00	82.85	6.51	51.21
**4m**	38.00	4.00	88.31	22.18	64.47
**4n**	27.00	6.00	57.14	55.13	23.92
**4o**	25.00	6.00	38.22	33.34	50.82
**4p**	85.00	1.00	62.60	16.51	39.28
**4q**	87.00	53.00	−5.19	25.19	93.11
**4r**	25.00	5.00	15.58	7.66	71.91
**4s**	54.00	4.00	79.16	30.39	28.85
**4t**	45.00	44.00	45.45	nd	46.63
**4u**	10.00	55.00	41.23	28.12	67.45
**4v**	64.00	15.00	6.49	1.60	24.88
**4w**	61.00	24.00	47.06	23.33	44.92
**4x**	48.00	47.00	8.16	29.45	63.76
**chlorothalonil**	69.93	56.27	12.12	53.40	39.41

Nd: not determined.

**Table 2:** The half maximal effective concentration (EC_50_) (μg mL^−1^) of the compounds **4b**.

Compound	Fungi	*EC_50_*
**4b**	*S. fuligenea*	11.2287
**flusilazole**	*S. fuligenea*	0.8923

**Table 3:** The structures, activities and total score of compounds.

No.	R	*D*	*D*″	*Residue*
**4a**	cycloprane	−1.53744	−1.6016	0.06416
**4b**	phenyl	−2.10898	−2.2012	0.09222
**4c**	*p*-nitro phenyl	−2.15762	−2.1771	0.01948
4d*	*p*-chloro phenyl	−0.6748	−0.6752	0.0004
**4e**	*p*-fluoro phenyl	−2.21145	−2.2910	0.07955
**4f**	*m*-methyl phenyl	−2.60927	−2.5781	−0.03117
**4g**#	*m*-chloro phenyl	−2.26605	−2.3169	0.05085
**4h**	*o*-F phenyl	−3.15846	−3.0991	−0.05936
**4i**	*o*-chloro phenyl	−3.03008	−2.9571	−0.07298
**4j**	2,4-dichloro phenyl	−2.72994	−2.80212	0.07218
**4k**	*o*-methoxyl phenyl	−2.32753	−2.4336	0.10607
**4l**	*p*-OMe Ph	−2.54624	−2.6018	0.05556
**4m**	*p*-iodo phenyl	−2.40876	−2.5266	0.11784
**4n**	isoxazoyl	−3.03926	−3.1169	0.07764
**4o**	1-cycan-cyclopropyl	−2.50183	−2.4923	−0.00953
**4p**	propyl	−2.67369	−2.5563	−0.11739
**4q**	Iso-propyl	−1.35375	−1.3239	−0.02985
**4r**	Butyl	−2.0958	−2.1276	0.0318
**4s**	2,4-dichlorophenoxymethyl	−3.03359	−3.1659	0.13231
**4t**#	[2-(2,4-dichlorophenoxy)acetyl]propyl	−2.71389	−2.6361	−0.07779
**4u**	furan	−2.20095	−2.3172	0.11625
**4v**	(2*E*,4*Z*)-hex	−2.99734	−3.1581	0.16076
**4w**	3-pyridine	−2.62024	−2.7768	0.15656
**4x**#	4-pyridine	−2.28632	−2.4019	0.11558

*D*: Experimental value,

*D*″: predictive value of *D*,

* template molecule,

# test.
